# Seasonal variation of pediatric asthma exacerbations and its association with asthma phenotypes

**DOI:** 10.1038/s41390-025-04073-2

**Published:** 2025-05-07

**Authors:** Firdian Makrufardi, Desy Rusmawatiningtyas, Indah Kartika Murni, Eggi Arguni, Yuan-Chien Lin, Kin-Fai Ho, Kian Fan Chung, Sheng-Chieh Lin, Hsiao-Chi Chuang

**Affiliations:** 1 International Ph.D. Program in Medicine, College of Medicine, Taipei Medical University, Taipei, Taiwan; 2 Department of Child Health, Faculty of Medicine, Public Health, and Nursing, Universitas Gadjah Mada – Dr. Sardjito Hospital, Yogyakarta, Indonesia; 3 Department of Civil Engineering, National Central University, Taoyuan City, Taiwan; 4 JC School of Public Health and Primary Care, The Chinese University of Hong Kong, Hong Kong, China; 5 National Heart and Lung Institute, Imperial College London, London, UK; 6 Department of Pediatrics, School of Medicine, College of Medicine, Taipei Medical University, Taipei, Taiwan; 7 Division of Allergy, Asthma, and Immunology, Department of Pediatrics, Shuang Ho Hospital, Taipei Medical University, New Taipei City, Taiwan; 8 School of Respiratory Therapy, College of Medicine, Taipei Medical University, Taipei, Taiwan; 9 Division of Pulmonary Medicine, Department of Internal Medicine, Shuang Ho Hospital, Taipei Medical University, New Taipei City, Taiwan; 10 Cell Physiology and Molecular Image Research Center, Wan Fang Hospital, Taipei Medical University, Taipei, Taiwan; 11 Graduate Institute of Medical Sciences, College of Medicine, Taipei Medical University, Taipei, Taiwan

## Abstract

**Background:**

The objective of this study was to examine the associations of blood inflammatory phenotypes with acute pediatric asthma exacerbations during different seasons and the COVID-19 pandemic.

**Methods:**

A retrospective study was conducted involving 32,160 pediatric asthma patients from January 2008 to December 2021. Asthma blood inflammatory phenotypes were categorized based on low (L) and high (H) eosinophils (E) and neutrophils (N) (LBE/HBE: ≥ 0.25 × 10^9^/L and LBN/HBN: ≥ 5 × 10^9^/L, respectively) and logistic regression was used to examine the odds ratio (OR) of outcome variables.

**Results:**

A 10^9^/L increase of neutrophils and eosinophils was associated with a 1.015-fold (95% CI: 1.009–1.021) and a 1.057-fold increase in the OR (95% CI: 1.026–1.088) for asthma exacerbations of hospitalized pediatric asthma patients. An increase in HBE/LBN phenotype was associated with a respective 1.232-fold (95% CI: 1.081–1.404) and 1.248-fold (95% CI: 1.101–1.414) increase in the OR for asthma exacerbations of hospitalized pediatric asthma patients before the COVID-19 pandemic in the winter and autumn seasons. However, an increase of LBE/LBN phenotype was associated with a respective 0.873-fold (95% CI: 0.769–0.991), 0.872-fold (95% CI: 0.771–0.986), and 0.813-fold (95% CI: 0.709–0.932) decrease in the OR for asthma exacerbations in the winter, spring and summer seasons.

**Conclusions:**

HBE/LBN phenotype had a higher risk of asthma exacerbations among hospitalized pediatric asthma patients in the winter and autumn, while LBE/LBN phenotype had a lower risk in the winter, spring, and summer.

**Impact:**

- Blood eosinophils and neutrophils have been indicated to have a potential influence on pediatric asthma development and severity.
- HBE/LBN phenotype was associated with increased asthma exacerbations among hospitalized pediatric asthma patients during winter and autumn.
- Eosinophil and neutrophil predominance exhibited a higher influence on pediatric asthma exacerbations.

## Introduction

Childhood asthma is a prevalent chronic respiratory condition that affects a significant proportion of the global pediatric population, with an estimated 10%–15% of children experiencing symptoms of the disease.^1^ Asthma is a multifaceted and highly diverse disease that includes a range of phenotypes, despite the fact that its typical clinical symptoms consist of wheezing, coughing, chest tightness, and shortness of breath.^2^ It is well established that blood inflammatory biomarker is an important factor for asthma severity.^3,4^ In addition, seasonal fluctuations significantly contribute to elevated air pollution and pollen, thereby promoting respiratory conditions such as airway inflammation and heightened lung responsiveness.^5^ A previous study found that the peak of the asthma exacerbations was in early fall and the beginning of the winter season.^6^ Population-based epidemiological studies have shown that some groups within general populations are more prone to experiencing such cold-weather-related symptoms.^7^ These underscore the importance of pediatric asthma blood inflammatory phenotypes, particularly their distinct clinical presentations across seasons, for more effective disease management.

Eosinophilic inflammation affects nearly half of pediatric asthma cases, correlating with increased disease severity, exacerbation frequency, and symptom burden.^8^ While eosinophils and neutrophils are important in asthma pathogenesis, research has predominantly focused on eosinophilic, severe T helper 2 (Th2) asthma.^9^ The rising focus on non-eosinophilic and non-inflammatory asthma relies on eosinophil and neutrophil markers derived from induced sputum; however, blood eosinophils and neutrophils offer greater accessibility across age groups and symptomatic statuses within extensive populations.^10^ A study involving 15,019 asthma patients identified that individuals exhibiting a high blood eosinophils and low blood neutrophils phenotype (HBE/LBN) had an increased odds ratio of 1.31 for experiencing more severe asthma symptoms.^11^ Eosinophil levels hold predictive value for therapeutic responsiveness in pediatric asthma management.^12^ Taken together, understanding the role of inflammatory cells in pediatric asthma phenotyping is crucial for the implementation of preventive measures in pediatric asthma.

COVID-19 has impacted the pattern of asthma emergency department visit and hospitalization.^13^ Several countries reported a substantial reduction in asthma exacerbations associated with COVID-19 pandemic-related restrictions.^14^ A previous study highlighted an overall reduction in asthma exacerbation rate by 33% across all demographic groups during the COVID-19 pandemic.^15^ Additionally, meteorological conditions and air pollution levels were significantly lower during the pandemic, leading to a decrease in pediatric asthma emergency department visits.^16^ Implementation of lockdown measures, encouraging people to stay indoors and limit outdoor activities, has led to reduced exposure to air pollutants and pollen, subsequently lowering pediatric asthma exacerbations.^17^ Overall, the COVID-19 pandemic has influenced the pattern of asthma exacerbations, highlighting the interplay between environmental factors and respiratory health outcomes.

Recent studies have indicated the potential influence of blood eosinophils and neutrophils on pediatric asthma development and severity. However, there remains a gap in understanding the association between asthma blood phenotypes and asthma exacerbations. Therefore, this study aimed to investigate the associations of asthma blood phenotypes with acute asthma exacerbations across various seasons and the COVID-19 pandemic.

## Materials and methods

### Ethical considerations

The study procedures followed an approved protocol authorized by the Taipei Medical University Joint Institutional Review Board (TMU-JIRB No N202307036). All procedures were conducted in accordance with the approved protocol. Given the retrospective design of this study and the use of anonymized data, the requirement for obtaining informed consent was waived, as it did not impact the clinical care process.

### Study design and data source

This study employed clinical data from the Taipei Medical University Clinical Research Database (TMUCRD), which compiles comprehensive clinical information sourced from Taipei Medical University Hospital, Wan Fang Hospital, and Shuang Ho Hospital. The database includes structured data (e.g., basic demographics, cause of death, medical history, cancer registry status, vital signs, pain scores, laboratory results, diagnoses, treatment procedures, surgery details, medication status and medical device usage) and unstructured data (e.g., physician notes, pathology reports, image examinations and radiology reports). The diagnoses recorded in the TMUCRD were determined in accordance with the diagnostic criteria specified in the International Classification of Diseases, Ninth and Tenth Revisions, Clinical Modification (ICD-9-CM and ICD-10-CM), respectively.

### Data collection

**Fig. 1:**
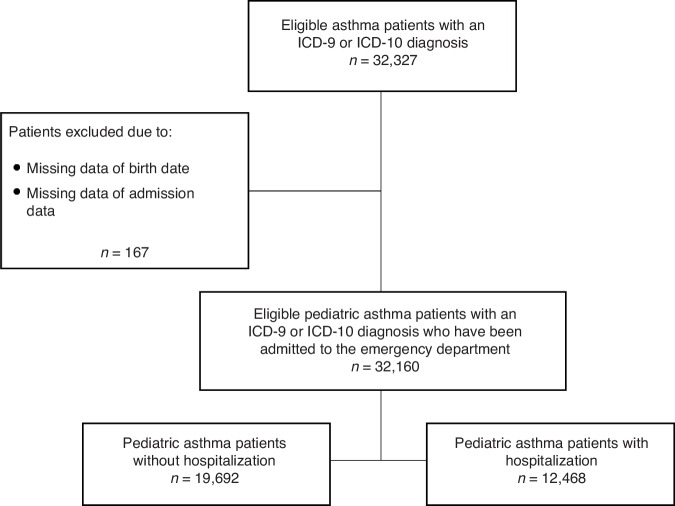
Flowchart of pediatric asthma patients.

A retrospective study involving 32,160 patients was conducted from January 2008 to December 2021 (Fig. 1). The selected features in the dataset from TMUCRD included: (1) demographic information: sex, age, and body mass index (BMI); (2) time of emergency department visits (season and COVID-19 period); (3) hospitalization status; and (4) laboratory test results: neutrophil and eosinophil. The inclusion criteria for this study consisted of subjects aged ≤18 years who had been admitted to the emergency department with asthma, underwent a blood test during admission, and were diagnosed with the ICD-9-CM code 493 and the ICD-10-CM code J45, respectively. The diagnosis of asthma was based on the Global Initiative for Asthma (GINA) guideline on a combination of medical history, physical examination, and supporting examination.^18,19^ The exclusion criteria consisted of subjects who were newly registered and had not previously sought medical care at the three hospitals due to the lack of complete medical background information records, or had been confirmed with chronic obstructive pulmonary disease, bronchiectasis, malignancy, or any chronic inflammatory condition unrelated to asthma. Asthma exacerbations were defined as the use of antibiotic treatment and admission to the hospital in asthma patients.^20^ The COVID-19 pandemic period was determined by the World Health Organization (WHO) declaration of a global pandemic in March 11, 2020.^21^ Meteorological and air pollution data in Northern Taiwan were accessed from the Central Weather Bureau (CWB) stations for daily-scale relative humidity and temperature, while data for hourly-scale PM_2.5_ concentration were obtained from the Environmental Protection Administration (EPA). The meteorological and air pollution data were rearranged and cleaned before being converted to monthly and seasonal average values.

### Blood inflammatory phenotypes

A white blood cell (WBC), neutrophil, and eosinophil count were determined for each participant by an accredited laboratory of the hospitals. We utilized previously established cut-off values for neutrophils and eosinophils to categorize asthma blood inflammatory phenotypes.^10,11,22^ Briefly, four asthma blood inflammatory phenotypes were defined, including low blood eosinophils to low blood neutrophils (LBE/LBN: eosinophils <0.25 × 10^9^/L and neutrophils <5 × 10^9^/L), low blood eosinophils to high blood neutrophils (LBE/HBN: eosinophils <0.25 × 10^9^/L and neutrophils ≥5 × 10^9^/L), high blood eosinophils to low blood neutrophils (HBE/LBN: eosinophils ≥0.25 × 10^9^/L and neutrophils <5 × 10^9^/L), and high blood eosinophils to high blood neutrophils (HBE/HBN: eosinophils ≥0.25 × 10^9^/L and neutrophils ≥5 × 10^9^/L).

### Statistical analysis

Continuous data were represented by mean and standard deviation (SD), while categorical data were expressed in counts and percentages. Employing a winsorization strategy, outliers were identified and mitigated by substituting values beyond the 1st and 99th percentiles.^23^ A normality test was conducted to assess the normal distribution of the data. Logistic regression was used to examine the odds ratio (OR) of outcome variables (asthma exacerbation) between the case (hospitalized patients) and control group (non-hospitalized patients). The Hosmer-Lemeshow test was utilized to evaluate the adequacy of fit for the logistic regression model. Patient characteristics, including age, sex, BMI, allergic rhinitis, and atopy, were adjusted for in the models to control for their potential confounding effects. Seasons were categorized as winter (December–February), spring (March–May), summer (June–August), and autumn (September–November). All analysis was conducted using SPSS for Windows version 26.0 (SPSS Inc., Chicago, IL). Statistical significance was determined based on a *P*-value < 0.05.

## Results

### Characteristics of study subjects

Table 1 summarizes the characteristics of the 32,160 patients recruited in this study, including 19,692 pediatric asthma patients without hospitalization and 12,468 pediatric asthma patients with hospitalization. The majority of patients were male (52.8%), and the average age was 4.9 years. The average BMI was 15.8 kg/m^2^. Among the participants, 41.3% had allergic rhinitis and 1.6% had atopy. The pediatric asthma hospitalization group had higher exacerbations in the winter (25.4%) and autumn (28.1%), while the non-hospitalization group had higher exacerbations in the summer (21.3%) and spring (30.6%) seasons. The asthma phenotypes showed that the LBN/LBE group had the highest exacerbations in both hospitalization and non-hospitalization groups (32.4% and 34.6%, respectively), followed by LBN/HBE (31.4% and 28.3%, respectively), HBN/LBE (24.1% and 21.6%, respectively), and HBN/HBE (12.1% and 15.5%, respectively). The hospitalized patients exhibited elevated counts of white blood cells, neutrophils, and eosinophils in comparison to the non-hospitalized patients (*p* < 0.05). The frequency of emergency department visits exhibited a higher prevalence before the onset of COVID-19 in comparison to the period during the COVID-19 pandemic (*p* < 0.05).

**Table 1:** Characteristics of pediatric patients with asthma emergency department visits.

Variables	Hospitalization (*n* = 12468)	Non-Hospitalization (*n* = 19692)	*p*-value
**Age (years), mean ± SD**	4.70 ± 3.47	5.05 ± 3.47	<0.05
**Sex,** ***n*** **(%)**			
Male	7366 (59.1)	11,984 (60.9)	<0.05
Female	5102 (40.9)	7708 (39.1)	
**BMI, mean ± SD**	15.78 ± 1.03	15.90 ± 1.24	<0.05
**Allergic rhinitis,** ***n*** **(%)**	5178 (41.5)	8110 (41.2)	<0.05
**Atopy,** ***n*** **(%)**	322 (1.6)	207 (1.7)	<0.05
**Hematological parameters, mean** **±** **SD**			
White blood cell, 10^9^/L	33.43 ± 67.43	20.48 ± 47.23	<0.05
Neutrophil, 10^9^/L	4.61 ± 5.27	4.21 ± 4.93	<0.05
Eosinophil, 10^9^/L	0.52 ± 1.11	0.47 ± 0.93	<0.05
**Asthma phenotypes,** ***n*** **(%)**			
LBE/LBN	2559 (32.4)	3644 (34.6)	<0.05
HBE/LBN	2480 (31.4)	2979 (28.3)	
LBE/HBN	1900 (24.1)	2279 (21.6)	
HBE/HBN	956 (12.1)	1639 (15.5)	
**Seasons,** ***n*** **(%)**			
Winter	3172 (25.4)	4845 (24.6)	<0.05
Autumn	3498 (28.1)	4629 (23.5)	
Summer	2593 (20.8)	4194 (21.3)	
Spring	3205 (25.7)	6024 (30.6)	
**COVID-19 pandemic status,** ***n*** **(%)**			
Before COVID-19 pandemic	19,557 (99.3)	12,250 (98.3)	<0.05
During COVID-19 pandemic	135 (0.7)	218 (1.7)	

*BMI* body mass index, *ED* emergency department, *LBE* low blood eosinophil, *LBN* low blood neutrophil, *HBE* high blood eosinophil, *HBN* high blood neutrophil.

### Associations of asthma phenotypes with asthma exacerbations

Associations of asthma phenotypes with asthma exacerbations are shown in Table 2. We observed that a 10^9^/L increase in white blood cells was associated with a 1.003-fold increase in the OR for asthma exacerbations of hospitalized pediatric asthma patients (95% CI: 1.003–1.003). A 10^9^/L increase in neutrophils was associated with a 1.015-fold increase in the OR for asthma exacerbations of hospitalized pediatric asthma patients (95% CI: 1.009–1.021). A 10^9^/L increase in eosinophils was associated with a 1.057-fold increase in the OR for asthma exacerbations of hospitalized pediatric asthma patients (95% CI: 1.026–1.088). An increase in HBE/LBN phenotype was associated with a 1.133-fold increase in the OR for asthma exacerbations of hospitalized pediatric asthma patients (95% CI: 1.062–1.209). However, an increase in LBE/LBN phenotype was associated with a 0.870-fold decrease in the OR for asthma exacerbations of hospitalized pediatric asthma patients (95% CI: 0.816–0.926).

**Table 2:** Associations (odds ratio (OR)) of hematology parameters and asthma phenotypes with asthma exacerbations between non-hospitalized pediatric asthma patients (reference group) and hospitalized pediatric asthma patients.

Characteristics	Crude OR (95% CI)	Adjusted OR (95% CI)
**Hematological parameters, 10**^**9**^**/L**		
White blood cell, 10^9^/L	1.003 (1.003–1.003)*	1.003 (1.003–1.003)*
Neutrophil, 10^9^/L	1.017 (1.011–1.023)*	1.015 (1.009–1.021)*
Eosinophil, 10^9^/L	1.061 (1.030–1.092)*	1.057 (1.026–1.088)*
**Asthma phenotypes, %**		
LBE/LBN, %	0.875 (0.822–0.932)*	0.870 (0.816–0.926)*
HBE/LBN, %	1.130 (1.059–1.205)*	1.133 (1.062–1.209)*
LBE/HBN, %	0.980 (0.913–1.052)	0.983 (0.916–1.056)
HBE/HBN, %	1.065 (0.978–1.159)	1.066 (0.979–1.160)

Model adjusted for age, sex, BMI, allergic rhinitis, and atopy.

*LBE* low blood eosinophil, *LBN* low blood neutrophil, *HBE* high blood eosinophil, *HBN* high blood neutrophil.

*p < 0.05. Values in bold characters are deemed statistically significant.

### Associations of asthma phenotypes with seasonal asthma exacerbations

Associations of asthma phenotypes with seasonal asthma exacerbations are shown in Table 3. We observed that a 10^9^/L increase in white blood cells, neutrophils, and eosinophils was associated with an increase in the OR for asthma exacerbations of hospitalized pediatric asthma patients in all seasons (*p* < 0.05). An increase in HBE/LBN phenotype was associated with a respective 1.214-fold, 1.166-fold and 1.239-fold increase in the OR for asthma exacerbations of hospitalized pediatric asthma patients in winter, summer and autumn seasons (95% CI: 1.067–1.381, 95% CI: 1.014–1.341 and 95% CI: 1.095–1.402, respectively). An increase in LBE/LBN phenotype was associated with a respective 0.873-fold, 0.872-fold and 0.813-fold decrease in the OR for asthma exacerbations of hospitalized pediatric asthma patients in winter, spring, and summer seasons (95% CI: 0.769–0.991, 95% CI: 0.771–0.986 and 95% CI: 0.709–0.932, respectively). An increase of LBE/HBN phenotype was associated with a 1.171-fold increase in the OR for asthma exacerbations in the spring season (95% CI: 1.020–1.343) and a 0.843-fold decrease in the autumn season (95% CI: 0.734–0.968) among hospitalized pediatric asthma patients.

**Table 3:** Associations (odds ratio (OR)) of hematology parameters and asthma phenotypes with seasonal asthma exacerbations between non-hospitalized pediatric asthma patients (reference group) and hospitalized pediatric asthma patients.

	Spring		Summer		Autumn		Winter	
	Crude OR (95% CI)	Adjusted OR (95% CI)	Crude OR (95% CI)	Adjusted OR (95% CI)	Crude OR (95% CI)	Adjusted OR (95% CI)	Crude OR (95% CI)	Adjusted OR (95% CI)
**Hematological parameters, 10**^**9**^**/L**								
White blood cell, 10^9^/L	1.003 (1.002–1.004)*	1.003 (1.002–1.003)*	1.003 (1.002–1.004)*	1.003 (1.002–1.004)*	1.003 (1.002–1.004)*	1.003 (1.002–1.004)*	1.003 (1.002–1.004)*	1.003 (1.002–1.004)*
Neutrophil, 10^9^/L	1.036 (1.024–1.048)*	1.035 (1.023–1.047)*	1.014 (1.001–1.026)*	1.010 (0.997–1.022)	1.003 (0.993–1.014)	1.002 (0.991–1.012)	1.017 (1.005–1.028)*	1.014 (1.002–1.026)*
Eosinophil, 10^9^/L	0.995 (0.928–1.066)	0.997 (0.930–1.068)	1.110 (1.047–1.177)*	1.109 (1.047–1.175)*	1.091 (1.036–1.149)*	1.082 (1.028–1.139)*	0.990 (0.922–1.062)	0.979 (0.911–1.052)
**Asthma phenotypes, %**								
LBE/LBN, %	0.873 (0.769–0.991)*	0.870 (0.766–0.988)*	0.872 (0.771–0.986)*	0.873 (0.772–0.987)*	0.813 (0.709–0.932)*	0.800 (0.697–0.918)*	0.936 (0.829–1.056)	0.930 (0.823–1.050)
HBE/LBN, %	1.214 (1.067–1.381)*	1.220 (1.072–1.388)*	0.943 (0.830–1.070)	0.940 (0.828–1.068)	1.166 (1.014–1.341)*	1.165 (1.013–1.340)*	1.239 (1.095–1.402)*	1.248 (1.103–1.413)*
LBE/HBN, %	0.894 (0.778–1.028)	0.895 (0.778–1.028)	1.171 (1.020–1.343)*	1.172 (1.022–1.345)*	1.061 (0.905–1.243)	1.076 (0.918–1.261)	0.843 (0.734–0.968)*	0.843 (0.734–0.968)*
HBE/HBN, %	1.089 (0.915–1.296)	1.035 (0.911–1.291)	1.124 (0.957–1.319)	1.124 (0.957–1.320)	1.034 (0.860–1.243)	1.045 (0.869–1.257)	0.992 (0.842–1.170)	0.992 (0.841–1.170)

Model adjusted for age, sex, BMI, allergic rhinitis, and atopy.

*OR* odds ratio, *CI* confidence interval, *LBE* low blood eosinophil, *LBN* low blood neutrophil, *HBE* high blood eosinophil, *HBN* high blood neutrophil.

*p < 0.05. Values in bold characters are deemed statistically significant.

### Associations of asthma phenotypes with asthma exacerbations during different COVID-19 periods

Associations of asthma phenotypes with asthma exacerbations during different COVID-19 periods are shown in Table 4. We observed that a 10^9^/L increase in white blood cells, neutrophils, and eosinophils was associated with an increase in the OR for asthma exacerbations of hospitalized pediatric asthma patients before the COVID-19 pandemic (*p* < 0.05). An increase in HBE/LBN phenotype was associated with a 1.142-fold increase in the OR for asthma exacerbations of hospitalized pediatric asthma patients (95% CI: 1.070–1.219). Moreover, an increase in LBE/LBN phenotype was associated with a 0.858-fold decrease in the OR for asthma exacerbations before the COVID-19 pandemic (95% CI: 0.805–0.914) and a 1.961-fold increase during the COVID-19 pandemic (95% CI: 1.027–3.742) of hospitalized pediatric asthma patients.

**Table 4:** Associations (odds ratio (OR)) of hematology parameters and asthma phenotypes with asthma exacerbations of non-hospitalized pediatric asthma patients (reference group) and hospitalized pediatric asthma patients in different COVID-19 periods.

	Before COVID-19 pandemic		During COVID-19 pandemic	
	Crude OR (95% CI)	Adjusted OR (95% CI)	Crude OR (95% CI)	Adjusted OR (95% CI)
**Hematological parameters, 10**^**9**^**/L**				
White blood cell, 10^9^/L	1.003 (1.003–1.003)*	1.003 (1.003–1.003)*	1.009 (0.987–1.032)	1.009 (0.987–1.032)
Neutrophil, 10^9^/L	1.016 (1.011–1.022)*	1.014 (1.009–1.020)*	1.138 (1.029–1.258)*	1.118 (1.005–1.244)*
Eosinophil, 10^9^/L	1.056 (1.026–1.088)*	1.056 (1.026–1.088)*	1.307 (0.871–1.961)	1.137 (0.832–1.554)
**Asthma phenotypes, %**				
LBE/LBN, %	0.865 (0.811–0.921)*	0.858 (0.805–0.914)*	1.873 (1.037–3.385)*	1.961 (1.027–3.742)*
HBE/LBN, %	1.137 (1.066–1.214)*	1.142 (1.070–1.219)*	0.682 (0.371–1.254)	0.624 (0.323–1.206)
LBE/HBN, %	0.988 (0.920–1.061)	0.992 (0.923–1.065)	0.517 (0.263–1.019)	0.576 (0.288–1.151)
HBE/HBN, %	1.063 (0.976–1.158)	1.065 (0.978–1.160)	1.597 (0.631–4.042)	1.587 (0.617–4.084)

Model adjusted for age, sex, BMI, allergic rhinitis, and atopy.

*OR* odds ratio, *CI* confidence interval, *LBE* low blood eosinophil, *LBN* low blood neutrophil, *HBE* high blood eosinophil, *HBN* high blood neutrophil.

*p < 0.05. Values in bold characters are deemed statistically significant.

### Associations of asthma phenotypes with seasonal asthma exacerbations during different COVID-19 periods

Associations of asthma phenotypes with seasonal asthma exacerbations during different COVID-19 periods are shown in Table 5. We observed that a 10^9^/L increase in white blood cells, neutrophils, and eosinophils was associated with an increase in the OR for asthma exacerbations of hospitalized pediatric asthma patients before the COVID-19 pandemic in all seasons (*p* < 0.05). An increase in HBE/LBN phenotype was associated with a respective 1.232-fold, 1.187-fold and 1.248-fold increase in the OR for asthma exacerbations of hospitalized pediatric asthma patients before the COVID-19 pandemic in winter, summer, and autumn seasons (95% CI: 1.081–1.404, 95% CI: 1.031–1.366, and 95% CI: 1.101–1.414, respectively). An increase in LBE/LBN phenotype was associated with a respective 0.858-fold, 0.862-fold and 0.786-fold decrease in the OR for asthma exacerbations of hospitalized pediatric asthma patients before the COVID-19 pandemic in winter, spring and summer seasons (95% CI: 0.755–0.976, 95% CI: 0.761–0.975, and 95% CI: 0.684–0.903, respectively). Moreover, an increase in LBE/HBN phenotype was associated with a 1.184-fold decrease in the OR for asthma exacerbations before the COVID-19 pandemic in the spring season (95% CI: 1.031–1.359) and 0.846-fold decrease in the autumn season (95% CI: 0.736–0.972), while 0.562-fold decrease in the summer seasons (95% CI: 0.340–0.929) during the COVID-19 pandemic of hospitalized pediatric asthma patients.

**Table 5:** Associations (odds ratio (OR)) of hematology parameters and asthma phenotypes with seasonal asthma exacerbations between non-hospitalized pediatric asthma patients (reference group) and hospitalized pediatric asthma patients in different COVID-19 periods.

	Spring		Summer		Autumn		Winter	
	Crude OR (95% CI)	Adjusted OR (95% CI)	Crude OR (95% CI)	Adjusted OR (95% CI)	Crude OR (95% CI)	Adjusted OR (95% CI)	Crude OR (95% CI)	Adjusted OR (95% CI)
**Before COVID-19 pandemic**								
**Hematological parameters, 10**^**9**^**/L**								
White blood cell, 10^9^/L	1.003 (1.002–1.004)*	1.003 (1.002–1.004)*	1.003 (1.002–1.004)*	1.003 (1.002–1.004)*	1.003 (1.002–1.004)*	1.003 (1.002–1.004)*	1.003 (1.002–1.004)*	1.003 (1.002–1.004)*
Neutrophil, 10^9^/L	**1.036 (1.024–1.049)**	1.036 (1.023–1.048)*	1.013 (1.000–1.025)*	1.009 (0.996–1.021)	1.003 (0.993–1.014)	1.002 (0.991–1.013)	1.015 (1.004–1.027)*	1.013 (1.001–1.025)*
Eosinophil, 10^9^/L	1.002 (0.934–1.074)	1.003 (0.935–1.076)	1.108 (1.045–1.175)*	1.107 (1.045–1.173)*	1.088 (1.033–1.147)*	1.080 (1.025–1.138)*	0.987 (0.919–1.059)	0.976 (0.908–1.049)
**Asthma phenotypes, %**								
LBE/LBN, %	0.861 (0.761–0.975)*	0.862 (0.761–0.975)*	0.802 (0.698–0.920)*	0.786 (0.684–0.903)*	0.926 (0.819–1.048)	0.918 (0.812–1.038)	0.863 (0.759–0.981)*	0.858 (0.755–0.976)*
HBE/LBN, %	0.946 (0.832–1.074)	0.944 (0.831–1.073)	1.186 (1.031–1.365)*	1.187 (1.031–1.366)*	1.237 (1.092–1.402)*	1.248 (1.101–1.414)*	1.224 (1.075–1.395)*	1.232 (1.081–1.404)*
LBE/HBN, %	1.183 (1.031–1.358)*	**1.184 (1.031–1.359**)*	1.076 (0.918–1.262)	1.094 (0.932–1.283)	0.846 (0.736–0.972)*	0.846 (0.736–0.972)*	0.901 (0.783–1.036)	0.901 (0.783–1.037)
HBE/HBN, %	1.124 (0.956–1.320)	1.125 (0.957–1.322)	1.008 (0.836–1.214)	1.019 (0.845–1.228)	1.012 (0.858–1.194)	1.012 (0.858–1.194)	1.083 (0.908–1.291)	1.080 (0.906–1.288)
**During COVID-19 pandemic**								
**Hematological parameters, 10**^**9**^**/L**								
White blood cell, 10^9^/L	1.041 (0.902–1.203)	1.026 (0.891–1.181)	0.867 (0.652–1.152)	0.790 (0.584–1.068)	1.009 (0.982–1.036)	1.011 (0.984–1.039)	0.911 (0.722–1.149)	0.855 (0.694–1.130)
Neutrophil, 10^9^/L	1.119 (0.740–1.693)	1.141 (0.717–1.817)	0.614 (0.411–0.916)*	0.562 (0.340–0.929)*	0.912 (0.807–1.032)	0.959 (0.836–1.099)	1.008 (0.875–1.160)	1.005 (0.867–1.165)
Eosinophil, 10^9^/L	2.028 (0.153–2.681)	1.058 (0.901–1.242)	1.352 (0.067–2.740)	1.629 (0.937–2.832)	1.400 (0.762–2.572)	1.394 (0.758–2.564)	0.619 (0.210–1.824)	0.586 (0.215–1.602)
**Asthma phenotypes, %**								
LBE/LBN, %	1.800 (0.487–6.649)	1.715 (0.392–7.498)	1.500 (0.414–5.436)	0.910 (0.208–3.977)	2.007 (0.676–5.961)	2.552 (0.753–8.648)	2.092 (0.646–6.779)	2.515 (0.636–9.945)
HBE/LBN, %	0.761 (0.181–3.197)	0.556 (0.102–3.035)	0.273 (0.062–1.203)	0.314 (0.065–1.514)	1.091 (0.383–3.108)	1.048 (0.347–3.164)	0.833 (0.257–2.703)	0.764 (0.205–2.849)
LBE/HBN, %	0.360 (0.084–1.542)	0.574 (0.113–2.913)	0.333 (0.049–2.271)	0.459 (0.062–3.383)	0.767 (0.206–2.856)	0.753 (0.201–2.831)	0.477 (0.153–1.491)	0.490 (0.152–1.581)
HBE/HBN, %	0.837 (0.572–1.224)	0.939 (0.672–1.312)	1.275 (0.832–1.953)	1.423 (0.931–2.175)	0.981(0.867–1.112)	0.972 (0.822–1.149)	1.267 (0.293–5.472)	1.317 (0.290–5.976)

Model adjusted for age, sex, BMI, allergic rhinitis, and atopy.

*OR* odds ratio, *CI* confidence interval, *LBE* low blood eosinophil, *LBN* low blood neutrophil, *HBE* high blood eosinophil, *HBN* high blood neutrophil.

*p < 0.05. Values in bold characters are deemed statistically significant.

## Discussion

The novelty of this study is that we investigated the associations of blood inflammatory phenotypes with acute exacerbations and hospital admissions in pediatric asthma patients across various seasons. We observed that the winter and autumn seasons increased the risk of acute exacerbations among hospitalized pediatric asthma patients. Also, we observed that periods before the COVID-19 pandemic were associated with increasing pediatric asthma exacerbations. These results suggest that pediatric asthma patients with HBE/LBN phenotypes may be particularly susceptible to seasonal variations and meteorological exposures.

We first observed that asthma phenotypes were associated with an increase in asthma exacerbations in hospitalized pediatric asthma patients. A previous study showed that an increase in eosinophil-predominant phenotypes increased the risk of pediatric asthma exacerbations by more than 2-fold (OR: 2.51, 95% CI: 1.12–5.59).^24^ Pediatric asthma patients were susceptible to virus infections due to the damage inflicted on epithelial cells by eosinophil-derived granule products.^25^ Eosinophils have been shown to promote viral clearance in the lung and contribute to innate immune responses against respiratory virus infections in humans.^26^ These mechanisms are mediated by the production of nitric oxide in response to the virus and Toll-like receptor (TLR) 7 to a synthetic agonist of the virus-sensing innate immune receptor.^27^ Additionally, eosinophils can suppress the expression of interferons and antiviral cytokines, resulting in an increased quantity during infections.^28^ We further observed the protective effect of low blood eosinophils and neutrophils in pediatric asthma exacerbations. A previous study demonstrated that treatment for normalizing eosinophil counts can help prevent asthma exacerbation.^29^ In addition, blood eosinophil counts were associated with exacerbation frequency and treatment in asthma patients.^12,30^ Taken together, the findings indicated that blood eosinophils play a pivotal role as the primary determinant of pediatric asthma exacerbations.

Our study further identified that asthma blood inflammatory phenotypes were associated with an increase in asthma exacerbations of hospitalized pediatric asthma patients during winter and autumn. A previous study showed that individuals with high eosinophil phenotypes were vulnerable to an increase in exacerbations during winter and autumn seasons.^31^ Eosinophils can produce cysLTs, serving as a significant cellular source of these mediators in the airways of individuals with seasonal allergic asthma, thereby contributing to airway remodeling.^32^ A previous study involving 32,143 Taiwanese school children showed that air pollution, with the exception of ozone, was associated with asthma exacerbations influenced by seasonal changes.^33^ In urban areas, pollution levels might be elevated during colder months, due to reduced dispersion of pollutants and increased energy consumption for heating.^34^ Moreover, we observed significant associations of LBE/LBN phenotype with a decrease in asthma exacerbations among hospitalized pediatric asthma patients. LBE/LBN phenotype has higher responsiveness to treatment, leading to improved asthma control and reduced exacerbation frequency.^35^ The findings suggest that seasonal variations may influence pediatric asthma exacerbations and treatment responsiveness across different asthma blood inflammatory phenotypes.

Additionally, we observed significant associations of HBE/LBN phenotype with an increase in asthma exacerbations before the COVID-19 pandemic. We also identified the meteorological and air pollution data for different months and seasons before and during the COVID-19 pandemic in Northern Taiwan, which can be seen in Supplementary Materials Figs. S1–S12. The periods before the COVID-19 pandemic exhibited higher concentrations of PM_2.5_, PM_10_, and NO_2_, and lower relative humidity percentages compared to periods during the COVID-19 pandemic. A previous study found a significant reduction in air pollution levels during the lockdown period, which contributed to a decreased incidence of pediatric asthma exacerbations.^36^ PM_2.5_ played an important role in pediatric asthma exacerbations and hospitalizations.^37^ PM_2.5_ stimulates heightened tissue kallikrein production in epithelial cells, leading to increased mucus secretion and enhanced barrier function, while also triggering elevated levels of immunoglobulin E and recruitment of eosinophils.^38^ Moreover, we observed a significant association between patients with high blood neutrophils and an increase in asthma exacerbations during the COVID-19 pandemic. Neutrophilic asthma patients have been shown to have broad lung function impairments, poor asthma control, and increased risk of severe exacerbations during the COVID-19 pandemic.^39^ Our results suggest that neutrophils had a more significant influence on pediatric asthma exacerbations during the COVID-19 pandemic period.

We further identified associations between asthma blood inflammatory phenotypes and an increase in pediatric asthma exacerbations before the COVID-19 pandemic during winter and autumn seasons. A previous study showed that patients with moderate-to-severe asthma and one or more exacerbations per month were more prevalent during the autumn-winter months before the COVID-19 pandemic.^40^ In addition, our study highlighted higher air pollution levels before the COVID-19 pandemic during colder seasons. Stable atmospheric conditions, such as temperature inversions, can lead to the trapping of pollutants near the surface, causing elevated levels of pollutants in the air during colder seasons.^41^ Meanwhile, we observed significant associations between neutrophil levels and the decrease in asthma exacerbations during the summer season amid the COVID-19 pandemic. During the summer months, characterized by warmer temperatures, there could be a dampening effect on viral transmission, thereby reducing the triggers for asthma exacerbations.^42^ Additionally, the implementation of public health measures, such as social distancing, mask-wearing, and enhanced hygiene practices, aimed at curbing the spread of COVID-19, may have inadvertently mitigated the transmission of other respiratory viruses known to exacerbate asthma.^43^ Together, our study suggests that the effects of asthma blood inflammatory phenotypes and seasonal variations became stronger with the increase in pediatric asthma exacerbations prior to the COVID-19 pandemic.

There are some limitations in this study. Firstly, potential confounding variables such as medication use (including inhaled corticosteroids, systemic corticosteroids, and biologics) were not accounted for, which could impact the study outcomes. Additionally, future investigations should take into account potential factors associated with exposure to air pollution and chemical compounds to provide a more comprehensive understanding of their influence on pediatric asthma exacerbations.

## Conclusion

In conclusion, our study highlights that the HBE/LBN phenotype was associated with increased asthma exacerbations among hospitalized pediatric asthma patients during the winter and autumn seasons. Furthermore, eosinophil and neutrophil predominance exhibited a higher influence on pediatric asthma exacerbations before the COVID-19 pandemic. These findings underscore the potential of asthma blood inflammatory phenotypes in predicting asthma control and management in clinical practice.

## Supplementary information

Supplementary information

## Data Availability

The datasets used and/or analyzed during the current study are available from the corresponding author on reasonable request.
