# Supplementary material for: Seasonal variation of pediatric asthma exacerbations and its association with asthma phenotypes
Source: Pediatr Res. 2025 May 7;98(6):2178–85. doi: 10.1038/s41390-025-04073-2 (PMC12811121; doi:10.1038/s41390-025-04073-2)
Supplement: Supplementary file 1 — Supplementary information [file 41390_2025_4073_MOESM1_ESM.pdf]

SUPPLEMENTARY INFORMATION

Seasonal variation of pediatric asthma exacerbations and its association with asthma phenotypes

Firdian Makrufardi[1,2], Desy Rusmawatiningtyas[2], Indah Kartika Murni[2], Eggi Arguni[2], Yuan-Chien Lin[3], Kin-Fai Ho[4], Kian Fan Chung[5], Sheng-Chieh Lin[6,7*], Hsiao-Chi Chuang[5,8,9,10,11*]

[1]International Ph.D. Program in Medicine, College of Medicine, Taipei Medical University, Taipei, Taiwan

[2]Department of Child Health, Faculty of Medicine, Public Health, and Nursing, Universitas Gadjah Mada – Dr. Sardjito Hospital, Yogyakarta, Indonesia

[3]Department of Civil Engineering, National Central University, Taoyuan City, Taiwan

[4]JC School of Public Health and Primary Care, The Chinese University of Hong Kong, Hong Kong, China

[5]National Heart and Lung Institute, Imperial College London, London, United Kingdom

[6]Department of Pediatrics, School of Medicine, College of Medicine, Taipei Medical University, Taipei, Taiwan

[7]Division of Allergy, Asthma, and Immunology, Department of Pediatrics, Shuang Ho Hospital, Taipei Medical University, New Taipei City, Taiwan

[8]School of Respiratory Therapy, College of Medicine, Taipei Medical University, Taipei, Taiwan

[9]Division of Pulmonary Medicine, Department of Internal Medicine, Shuang Ho Hospital, Taipei Medical University, New Taipei City, Taiwan

[10]Cell Physiology and Molecular Image Research Center, Wan Fang Hospital, Taipei Medical University, Taipei, Taiwan

[11]Graduate Institute of Medical Sciences, College of Medicine, Taipei Medical University, Taipei, Taiwan

***Corresponding Authors**

Sheng-Chieh Lin

Department of Pediatrics, School of Medicine, College of Medicine, Taipei Medical University, 250 Wuxing Street, Taipei 11031, Taiwan.

Telephone: +886-2-22490088 ext. 2951. Fax: +886-2-22490088. E-mail: jacklinbox@tmu.edu.tw

Hsiao-Chi Chuang

Inhalation Toxicology Research Lab (ITRL), School of Respiratory Therapy, College of Medicine, Taipei Medical University, 250 Wuxing Street, Taipei 11031, Taiwan.

Telephone: +886-2-27361661 ext. 3513. Fax: +886-2-27391143. E-mail: chuanghc@tmu.edu.tw

List of Figures

Figure S1. Monthly curves for the averages (%) of ambient relative humidity (RH) during two different periods: before the COVID-19 pandemic and during the COVID-19 pandemic in Northern Taiwan.

Figure S2. Seasonally curves for the averages (%) of ambient relative humidity (RH) during two different periods: before the COVID-19 pandemic and during the COVID-19 pandemic in Northern Taiwan.

Figure S3. Monthly curves for the averages (°C) of ambient temperature during two different periods: before the COVID-19 pandemic and during the COVID-19 pandemic in Northern Taiwan.

Figure S4. Seasonally curves for the averages (°C) of ambient temperature during two different periods: before the COVID-19 pandemic and during the COVID-19 pandemic in Northern Taiwan.

Figure S5. Monthly curves for the averages of nitrogen dioxide (NO_2) in ppb during two different periods: before the COVID-19 pandemic and during the COVID-19 pandemic in Northern Taiwan.

Figure S6. Seasonally curves for the averages of nitrogen dioxide (NO_2) in ppb during two different periods: before the COVID-19 pandemic and during the COVID-19 pandemic in Northern Taiwan.

Figure S7. Monthly curves for the averages of particulate matter with an aerodynamic diameter of <2.5 μm ($PM_{2.5}$) in μg/m^3 during two different periods: before the COVID-19 pandemic and during the COVID-19 pandemic in Northern Taiwan.

Figure S8. Seasonally curves for the averages of particulate matter with an aerodynamic diameter of <2.5 μm ($PM_{2.5}$) in μg/m^3 during two different periods: before the COVID-19 pandemic and during the COVID-19 pandemic in Northern Taiwan.

Figure S9. Monthly curves for the averages of particulate matter with an aerodynamic diameter of <10 μm (PM_{10}) in μg/m^3 during two different periods: before the COVID-19 pandemic and during the COVID-19 pandemic in Northern Taiwan.

Figure S10. Seasonally curves for the averages of particulate matter with an aerodynamic diameter of <10 μm (PM_{10}) in μg/m^3 during two different periods: before the COVID-19 pandemic and during the COVID-19 pandemic in Northern Taiwan.

Figure S11. Monthly curves for the averages of ozone (O_3) in μg/m^3 during two different periods: before the COVID-19 pandemic and during the COVID-19 pandemic in Northern Taiwan.

Figure S12. Seasonally curves for the averages of ozone (O_3) in μg/m^3 during two different periods: before the COVID-19 pandemic and during the COVID-19 pandemic in Northern Taiwan.

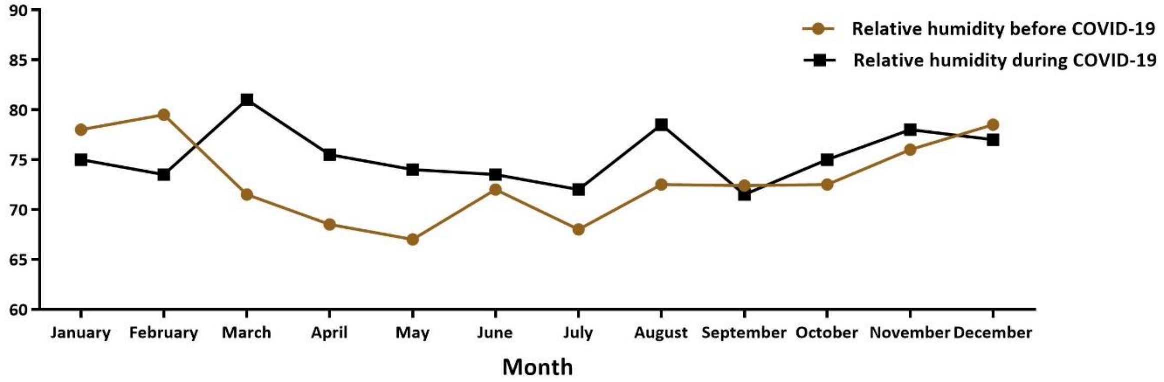

Figure S1. Monthly curves for the averages (%) of ambient relative humidity (RH) during two different periods: before the COVID-19 pandemic and during the COVID-19 pandemic in Northern Taiwan.

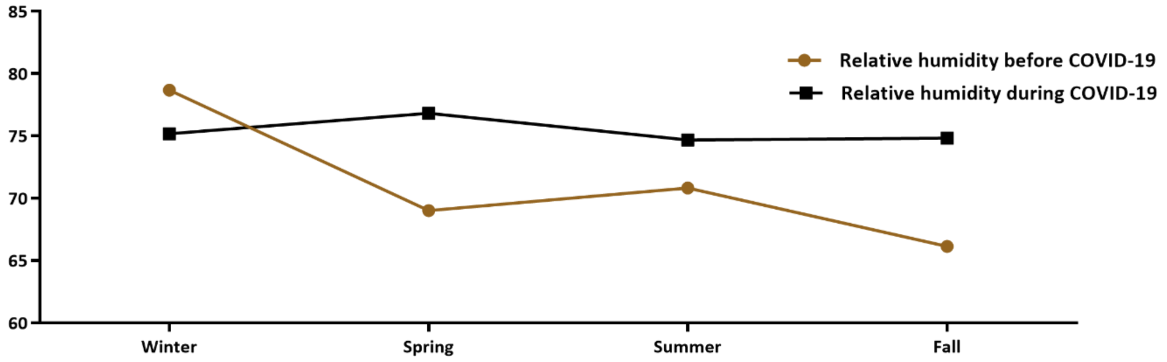

Figure S2. Seasonally curves for the averages (%) of ambient relative humidity (RH) during two different periods: before the COVID-19 pandemic and during the COVID-19 pandemic in Northern Taiwan.

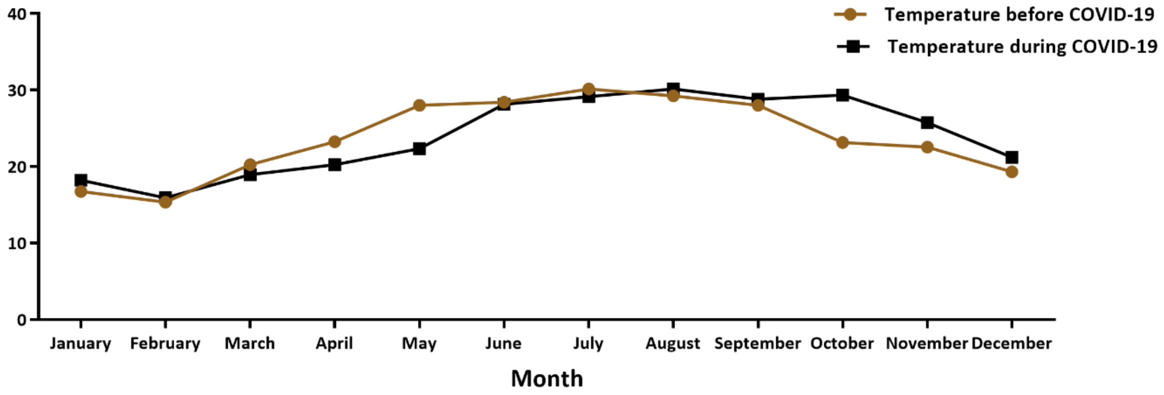

Figure S3. Monthly curves for the averages (°C) of ambient temperature during two different periods: before the COVID-19 pandemic and during the COVID-19 pandemic in Northern Taiwan.

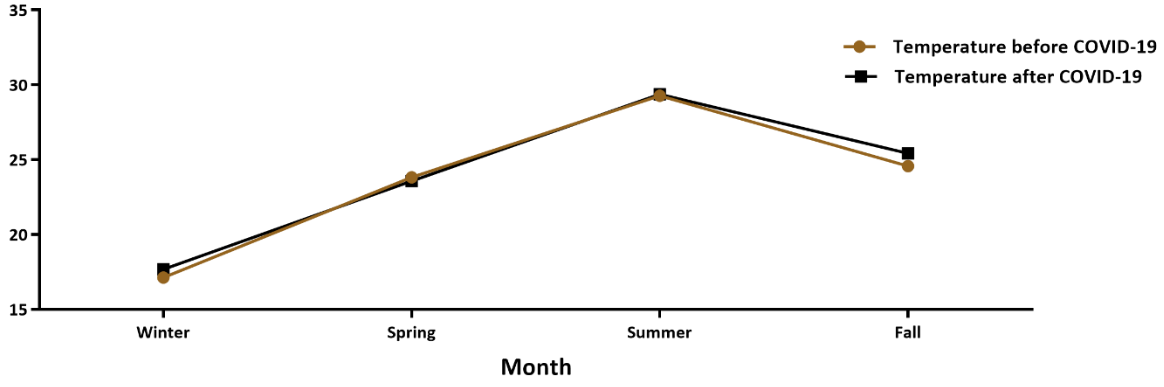

Figure S4. Seasonally curves for the averages (°C) of ambient temperature during two different periods: before the COVID-19 pandemic and during the COVID-19 pandemic in Northern Taiwan.

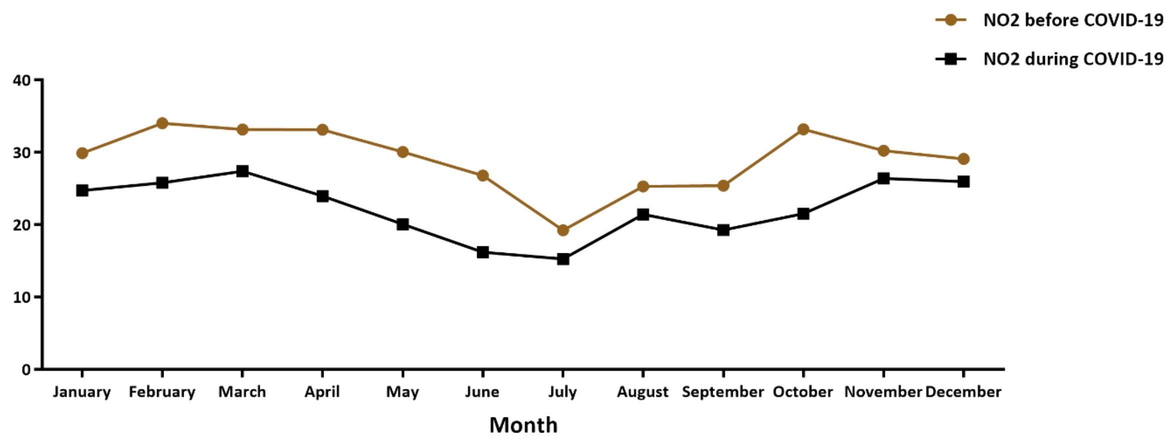

Figure S5. Monthly curves for the averages of nitrogen dioxide (NO_2) in ppb during two different periods: before the COVID-19 pandemic and during the COVID-19 pandemic in Northern Taiwan.

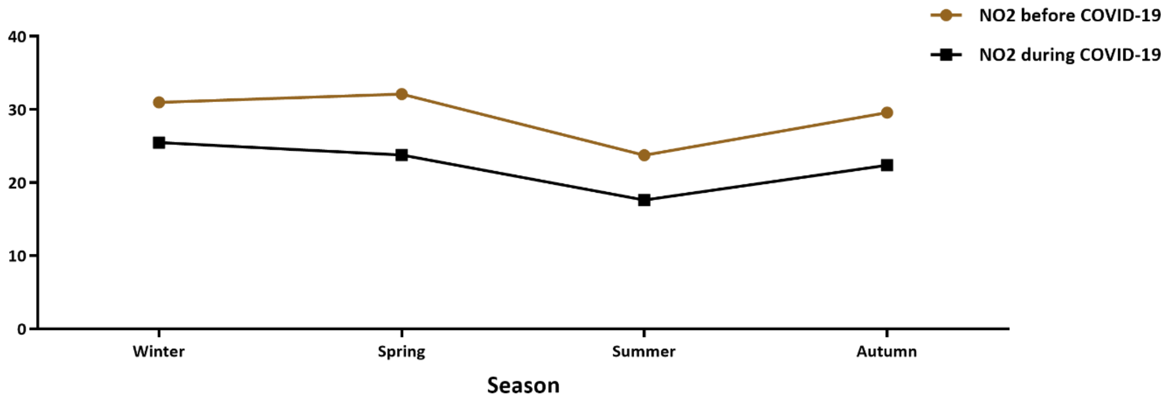

Figure S6. Seasonally curves for the averages of nitrogen dioxide (NO_2) in ppb during two different periods: before the COVID-19 pandemic and during the COVID-19 pandemic in Northern Taiwan.

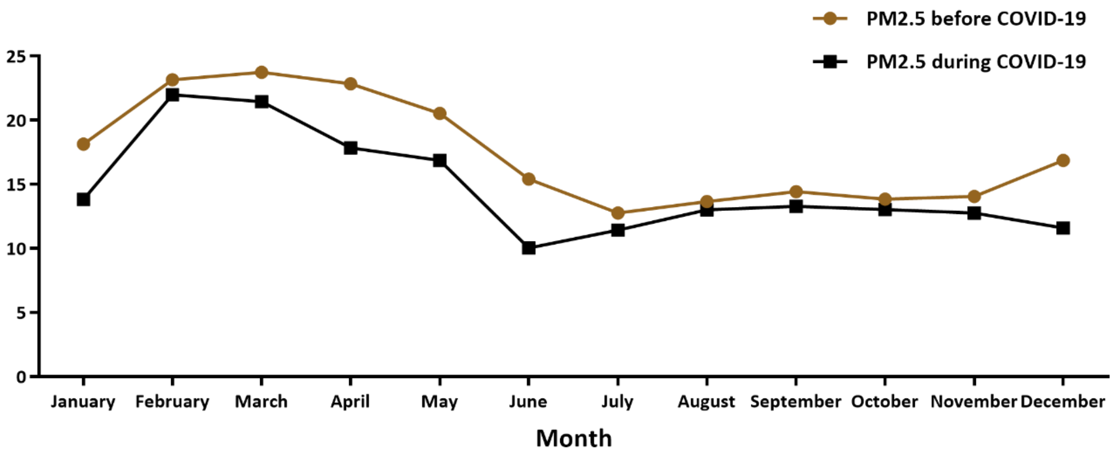

Figure S7. Monthly curves for the averages of particulate matter with an aerodynamic diameter of <2.5 μm ($PM_{2.5}$) in μg/m^3 during two different periods: before the COVID-19 pandemic and during the COVID-19 pandemic in Northern Taiwan.

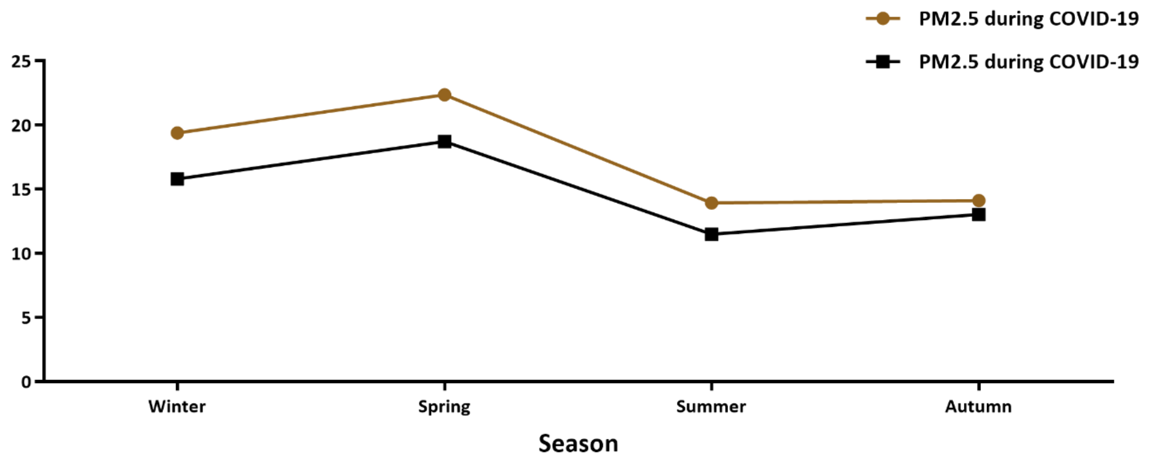

Figure S8. Seasonally curves for the averages of particulate matter with an aerodynamic diameter of <2.5 μm ($PM_{2.5}$) in μg/m^3 during two different periods: before the COVID-19 pandemic and during the COVID-19 pandemic in Northern Taiwan.

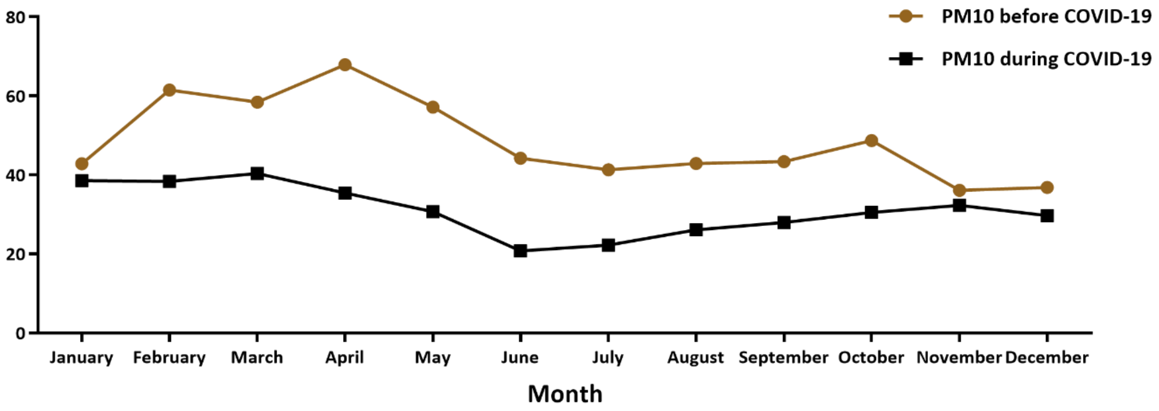

Figure S9. Monthly curves for the averages of particulate matter with an aerodynamic diameter of <10 µm (PM_{10}) in µg/m^3 during two different periods: before the COVID-19 pandemic and during the COVID-19 pandemic in Northern Taiwan.

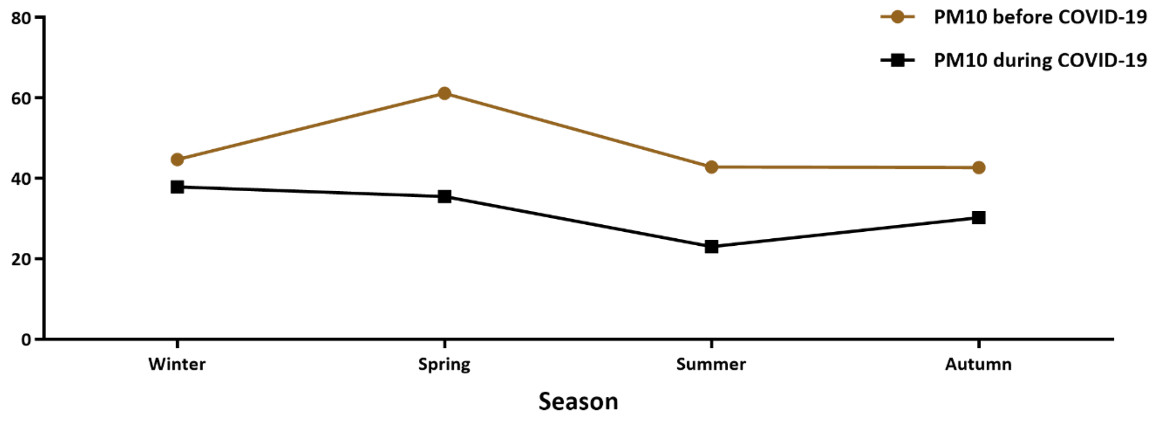

Figure S10. Seasonally curves for the averages of particulate matter with an aerodynamic diameter of <10 µm (PM_{10}) in µg/m^3 during two different periods: before the COVID-19 pandemic and during the COVID-19 pandemic in Northern Taiwan.

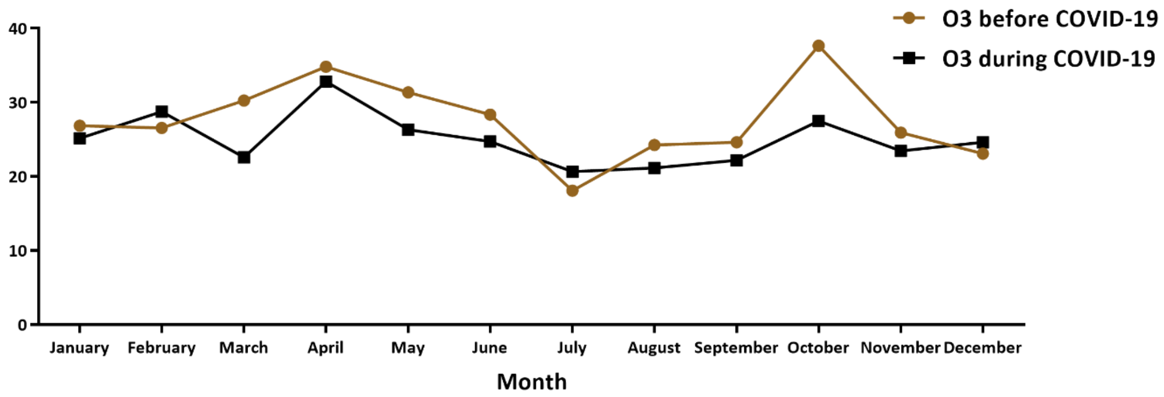

Figure S11. Monthly curves for the averages of ozone (O_3) in μg/m^3 during two different periods: before the COVID-19 pandemic and during the COVID-19 pandemic in Northern Taiwan.

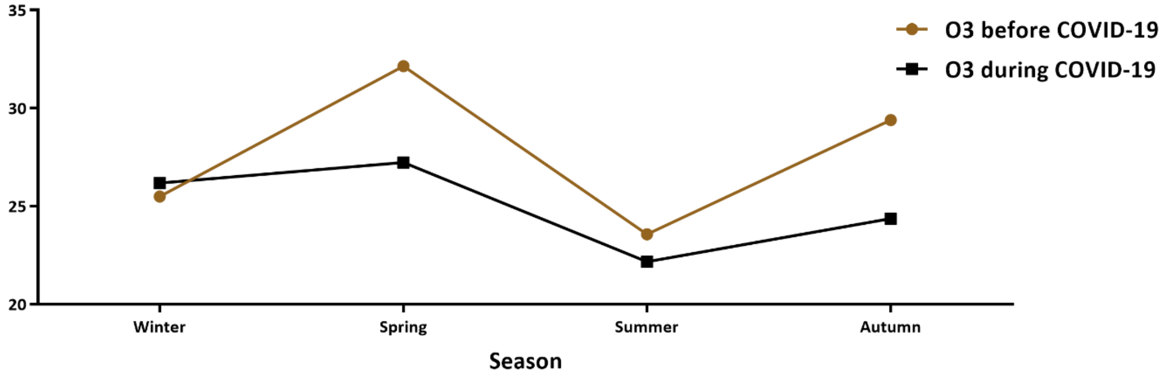

Figure S12. Seasonally curves for the averages of ozone (O_3) in μg/m^3 during two different periods: before the COVID-19 pandemic and during the COVID-19 pandemic in Northern Taiwan.
